# P-1011. Clostridioides difficile infection outcomes following implementation of 2-step testing at a large Veterans Affair hospital in South Texas

**DOI:** 10.1093/ofid/ofaf695.1208

**Published:** 2026-01-11

**Authors:** Maryem R Lodhra, Cindy Muyna, Lauren N Garza, Sean O’Neil, Jose Cadena-Zuluaga

**Affiliations:** UT HEALTH SAN ANTONIO, SAN ANTONIO, TX; South Texas Veterans Healthcare System, San Antonio, Texas; South Texas Veteran Health Care System, San Antonio, Texas; South Texas Veterans Affairs, San Antonio, Texas; South Texas Veterans Healthcare System, UT Health San Antonio, San Antonio, Texas

## Abstract

**Background:**

*Clostridioides difficile* is a significant pathogen in community-onset and healthcare facility-onset diarrhea. *Clostridioides difficile* infection (CDI) remains a diagnostic challenge in healthcare. Guidelines for *C. difficile* testing recommend a multistep algorithm (GDH + toxin, GDH + toxin arbitrated by NAAT, or NAAT + toxin) rather than NAAT testing alone. Our institution implemented 2-step *C. difficile* testing 01/08/2024, to reduce rates of hospital-onset *Clostridioides difficile* infections (HO-CDI). Our study aims to evaluate the CDI outcomes following implementation of 2-step testing at Audie L. Murphy Veterans Affair hospital in San Antonio, Texas.

**Methods:**

A multidisciplinary team monitoring HO-CDI compiled data of all positive *C. difficile* PCR results during the study period (January 8, 2024 to April 15, 2025). Two-step testing (GDH and toxin A/B EIA) was subsequently performed on patients with positive PCR results. Of the total positive PCR results we evaluated the number of cases with positive PCR and negative toxin EIA. We investigated how many patients were treated initially despite discordant results (positive PCR and negative toxin EIA). Additionally, of those patients with discordant results who were not treated initially we reviewed 1) how many required treatment at 28 days and 2) CDI associated mortality at 28 days.

**Results:**

There were 104 cases of positive *C. difficile* PCRs during the study period. 78 of the 104 cases were patients with discordant results (75%). 47 of the 78 cases (60.3%) were treated initially despite positive PCR and negative toxin A/B EIA. None of the 31 cases with discordant results, which were not treated initially, required treatment at 28 days. CDI associated mortality at 28 days in those patients with discordant results who were not treated initially was zero.

**Conclusion:**

Our study demonstrates the reassuring CDI outcomes at 28 days in patients who had discordant 2-step testing results (positive PCR and negative toxin EIA). There was zero CDI associated mortality rate at 28 days in those patients who were not treated initially. This highlights the importance of the implementation of 2-step testing for *Clostridioides difficile* to improve diagnostic stewardship and minimize unnecessary treatment in patients who may be colonized with *C. difficile.*

**Disclosures:**

All Authors: No reported disclosures

